# Superior Nasal Septal Deviation as a Contributing Factor for Dacryocystitis

**DOI:** 10.7759/cureus.75433

**Published:** 2024-12-09

**Authors:** Stefan Konsulov

**Affiliations:** 1 Department of Otorhinolaryngology, Medical University of Plovdiv, Plovdiv, BGR

**Keywords:** chronic dacryocystitis, concha bullosa, endoscopic dacryocystorhinostomy, epiphora, external dacryocystorhinostomy, nasal septal deviation, nasolacrimal duct obstruction

## Abstract

Background

Dacryocystitis (DC) is a disease most often caused by an obstruction of the nasolacrimal duct, leading to over-accumulation of tears in the lacrimal sac, epiphora, and aseptic inflammation. External and endoscopic dacryocystorhinostomy (DCR) aims to restore the tear pathway by creating a bypass from the lacrimal sac to the nose. The aim of this study is to investigate superior nasal septal deviation as a possible contributing factor in the incidence and treatment of dacryocystitis.

Methods

This retrospective study included 36 patients surgically treated for chronic dacryocystitis from September 2019 to September 2024.

Results

Twenty-eight out of the 36 studied patients (77.8%) were females. The average age of all patients was 66.8 years, with 69.4% being in the 50-70 years age group while 30.6% being older than 70 years. Superior nasal septal deviation was present in all patients, with 18 (50%) cases being towards the side of the DC and 11 (30.6%) away from the side of the DC. In seven cases (19.4%), the septum was S-shaped. In none of the cases in which the endoscopic approach was used was a septoplasty necessary. Concha bullosa was observed in five cases (13.9%) and was treated in all. A total of 32 patients were treated endoscopically (88.9%) and four (11.1%) by the Toti external approach method. The intraoperative microbiologic culture was positive in a total of nine cases (25%), five being coagulase-negative Staphylococci (13.9%), two being S. pneumoniae (2.8%), and one each of S. aureus (2.8%) and Pseudomonas maltophilia (5.6%). A re-stenosis was observed in five patients (13.9%).

Conclusion

Superior nasal septal deviation is an emerging factor both for the incidence of dacryocystitis and the development of re-stenosis after dacryocystorhinostomy. Further studies are needed to find the exact types of nasal septum deviations carrying the greatest risk for disease development, as well as the role of septoplasty in the result of treatment.

## Introduction

The lacrimal glands are exocrine serous glands, with the main gland located in a shallow depression along the superior lacrimal orbit [1]. Two canaliculi, first running perpendicular and then parallel to the lid margin, join to form a lacrimal sac located in the oval lacrimal fossa. The latter is formed by the frontal process of the maxilla anteriorly and the uncinated bone posteriorly, between the anterior and posterior lacrimal crests [2]. From the lacrimal sac begins the nasolacrimal canal, which is initially osseous, formed by the maxillary, lacrimal, and inferior turbinate bones, and then continues as a membranous canal for the final quarter of its length. The nasolacrimal canal ends as Hasner's valve in the inferior meatus, about one centimeter posterior to the anterior end of the inferior turbinate [3]. Hasner's valve is constructed of several variable mucous membrane folds and prevents anterograde reflux of secretions [1].

Dacryocystitis (DC) is a disease affecting the lacrimal drainage system, presenting with epiphora. Most often, an obstruction of the nasolacrimal duct is the culprit, leading to an over-accumulation of tears in the lacrimal sac and subsequent aseptic inflammation [1]. Additionally, septic inflammation may ensue.

Dacryocystitis may be congenital or acquired. Congenital DC affects 6-20% of newborn infants, with 80-96% of them self-resolving within the first year [4-8]. Acquired DC can be due to infectious, inflammatory, neoplastic, traumatic, and mechanical causes [1].

Dacryocystorhinostomy (DCR) is the procedure aiming to restore the tear pathway by creating a bypass from the lacrimal sac to the nose and could be external or endoscopic endonasal. Endoscopic DCR is minimally invasive and usually well-tolerated by patients, having many advantages, such as no external scar, less bleeding, and a smaller chance of injury to adjacent structures, such as the angular vein, preservation of the lacrimal pump function and faster overall recovery of patients [1,9-10].

## Materials and methods

This retrospective study includes 36 patients surgically treated for chronic dacryocystitis from September 2019 to September 2024 at the Department of Otorhinolaryngology at University Hospital Kaspela, Plovdiv, Bulgaria. All of the included patients had been treated previously unsuccessfully by an ophthalmologist and referred for dacryocystorhinostomy. The data was collected from electronic records and additionally included computed tomography and intraoperative endoscopic images. A descriptive and statistical analysis of the data was performed.

After receiving informed consent, the physical exam in all patients was performed by applying gentle pressure over the lacrimal sac, with mucopurulent reflux being suggestive of nasolacrimal obstruction. Following probing and syringing, an Anel lavage with an injection of less than 5 ml saline into the inferior lacrimal punctum, where a reflux was indicative of canalicular obstruction. Nasal endoscopy and computed tomography of the nose and paranasal sinuses were performed to search for possible endonasal etiologic factors such as concha bullosa and nasal septal deviation. For the purpose of this study, the superior nasal septal deviation was defined as a deviation on the level of the middle turbinate or higher on coronal computed tomography scans. If present, the deviation was recorded as being towards or away from the side with DC or S-shaped.

The external procedure employed was described by Toti and later modified by Dupuy-Dutemps and Bourguet [11].

**Figure 1:**
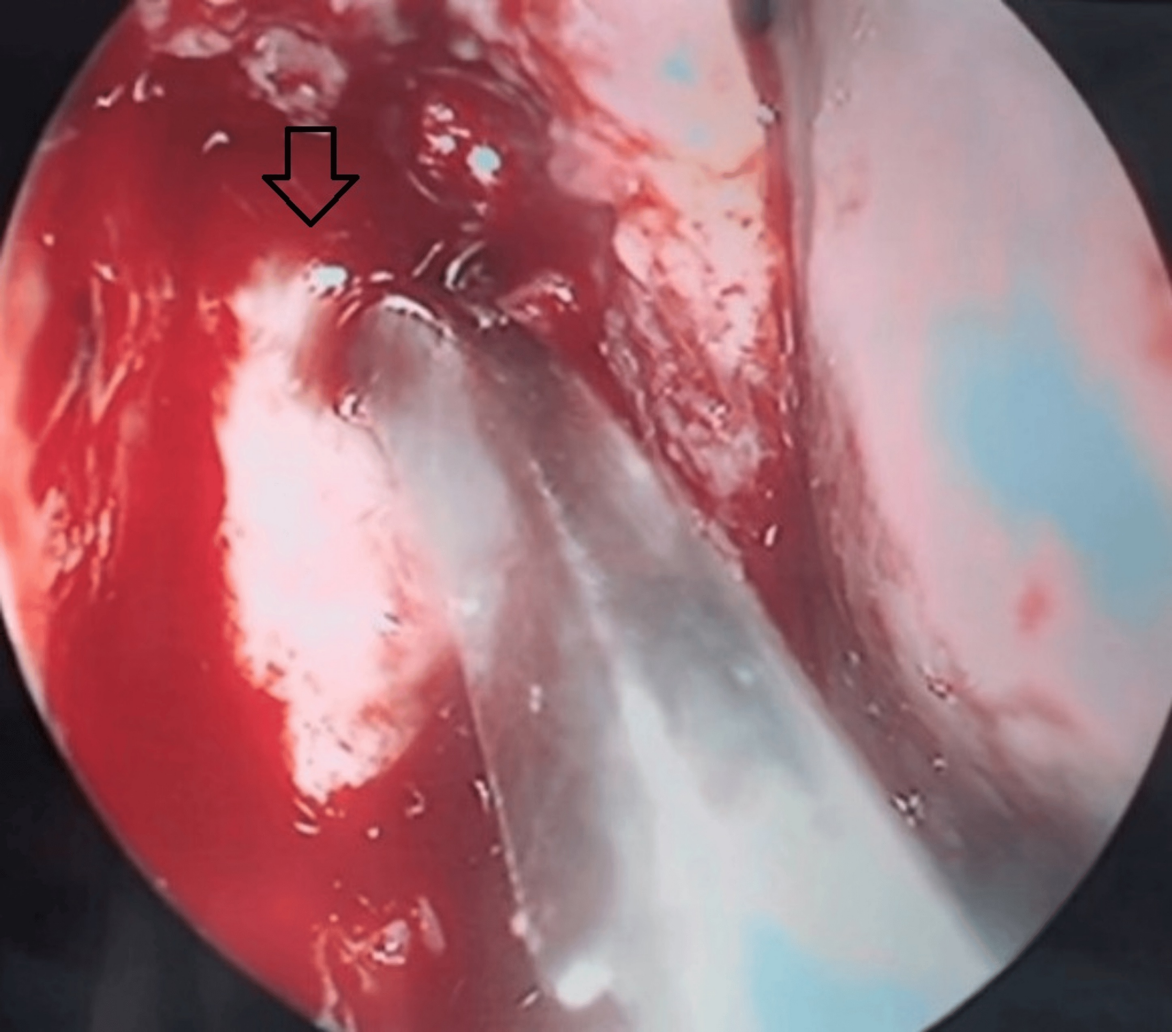
Right-sided endoscopic dacryocystorhinostomy using a sickle-shaped knife The arrow shows the right lacrimal sac.

The endoscopic endonasal procedure in all cases began with the infiltration of the ipsilateral lateral nasal wall with lidocaine and adrenaline (concentration 1:100,000). Following, monopolar needle cauterization was used, and a mucosal flap was created using a suction Freer elevator, presenting the frontal process of the maxilla and lacrimal bone. The Kerrison punch forceps were then used to present the lacrimal sac. The superior and inferior lacrimal punctums were then dilatated using a dilitator, after which an I-probe was used to reach the bottom of the lacrimal sac through the inferior lacrimal punctum. A sickle knife was then used to perform the marsupialization (Figure 1).

Following, using the Blakesley forceps, parts of the lateral and posterior walls of the lacrimal sac were removed, as well as a part of the mucosal flap. The eye was syringed with saline, and tobramycin/dexamethasone 3 mg/ 1 mg/ml eye drops were applied. The mucosal flap was then re-applied, and a nasal splint was placed together with a nasal pack for 24 hours.

## Results

Twenty-eight out of the 36 studied patients (77.8%) were females. The average age of all patients was 66.8 years, with 69.4% being in the 50-70 years age group while 30.6% being older than 70 years. The youngest patient was 53 years old, and the oldest was 80 years old. In 20 cases (55.6%), the DC was on the right side of the face, and in the rest (16 cases; 44.4%) was on the left.

**Figure 2:**
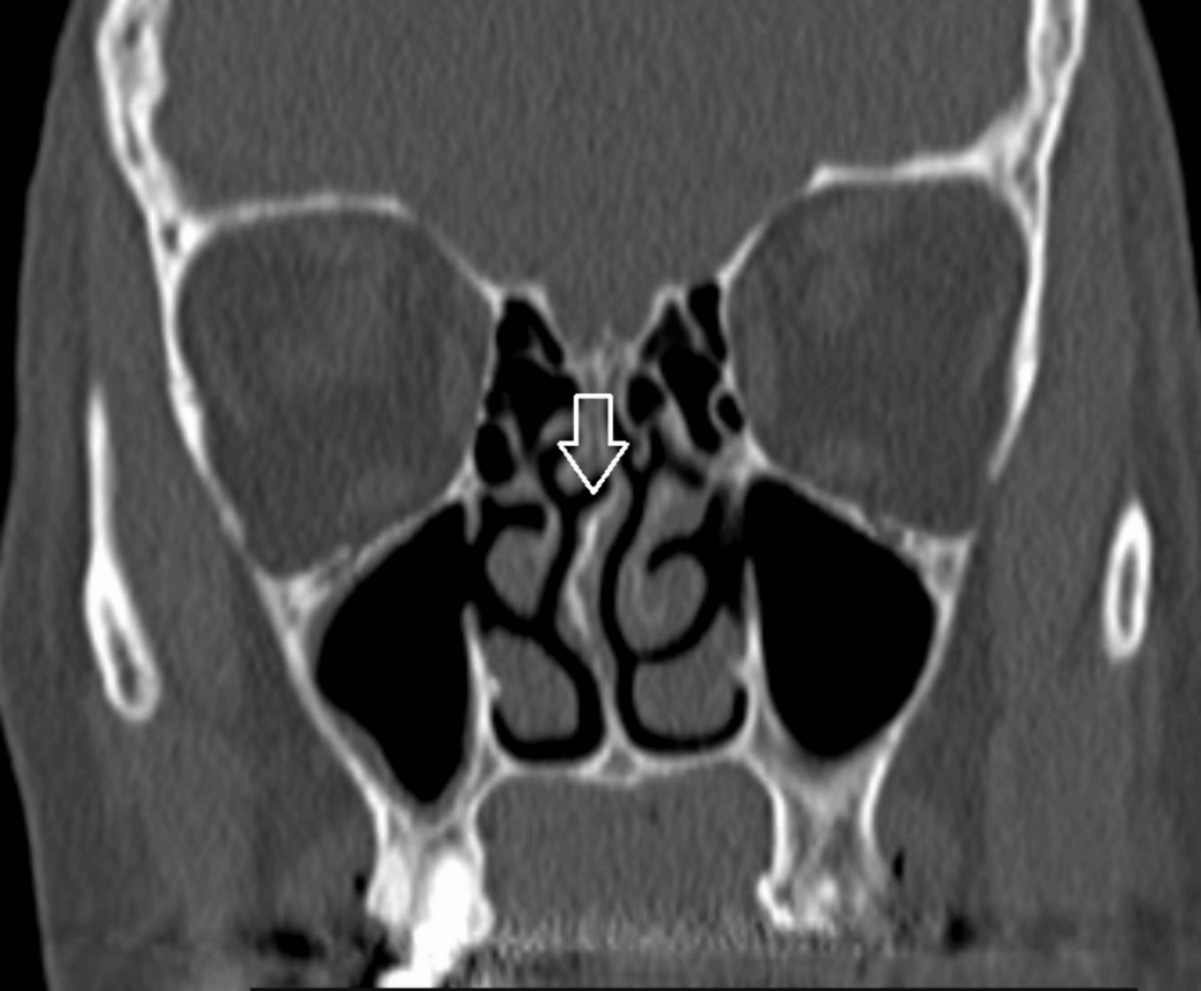
Superior nasal septal deviation towards the side of dacryocystitis

Superior nasal septal deviation was present in all patients, with 18 (50%) cases being towards the side of the DC (Figure 2) and 11 (30.6%) away from the side of the DC. In seven cases (19.4%), the septum was S-shaped. A septoplasty was not necessary in any of the cases where the endoscopic approach was used. Concha bullosa was observed in five cases (13.9%) and was treated in all.

A total of 32 patients were treated endoscopically (88.9%) and four (11.1%) by the Toti external approach method. Three of the patients treated externally (75%) were because of previous trauma and one (25%) was due to dacryolithiasis. Two of the patients treated externally, both of which had a history of trauma, were diagnosed with a re-stenosis - one at the second-week follow-up and one at the first-month postoperative follow-up. They were subsequently treated successfully endoscopically.

The intraoperative microbiologic culture was positive in a total of nine cases (25%), five being coagulase-negative Staphylococci (13.9%), two being S. pneumoniae (2.8%), and one each of S. aureus (2.8%) and Pseudomonas maltophilia (5.6%). The patients with a positive microbiologic culture were treated postoperatively according to the antibiogram results - seven patients (19.4%) with levofloxacin 5 mg/ml eye drops, one patient (2.8%) with betamethasone/gentamicin 1 mg/ml + 3 mg/ml eye drops, and one patient (2.8%) with oral ciprofloxacin 500 mg. The patient who received oral ciprofloxacin 500 mg had Pseudomonas maltophilia isolated. The patient who received betamethasone/gentamicin 1 mg/ml + 3 mg/ml eye drops has Staphylococcus aureus isolated. The patients who received levofloxacin 5 mg/ml eye drops had coagulase-negative Staphylococcus or Streptococcus pneumoniae isolated. The rest of the patients (75%), in which no microorganism was isolated, were continued with postoperative tobramycin/dexamethasone 3 mg/1 mg/ml eye drops for one week. The control microbiology samples at 14 days after treatment were negative in all patients in which a microorganism had been previously isolated.

A re-stenosis was observed in five patients (13.9%). Two of them (40%) had been treated by the Toti external approach, while the rest were treated endoscopically (60%). A silicone stent was subsequently placed, and no repeat re-stenosis was observed.

## Discussion

The lacrimal glands are exocrine serous glands, with the main gland being located along the superior lacrimal orbit [1]. Two canaliculi in the medial ocular canthus join to form a lacrimal sac, continuing as the nasolacrimal canal and draining the tears through Hasner's valve into the inferior meatus. Dacryocystitis is a congenital or acquitted inflammation of the lacrimal sac, most often due to an obstruction of the nasolacrimal duct and presenting with epiphora [1]. Our demographic data results show that acquired DC predominately affects females of advanced age.

Superior nasal septal deviation was present in all of the cases in this series and may be a factor in the obstruction of the nasolacrimal duct and DC development. Using a different classification system of nasal septum deviation based on severity, Chavadaki et al. found a significant association between nasal septal deviation and DC [12]. The present study is significant in its focus on deviation of the superior portion of the nasal septum, not severity. In a smaller portion of cases in this series, the superior portion of the nasal septum deviated away from the DC, underlining that nasal septal deviation is not the only factor contributing to the development of DC. Additionally, concha bullosa may play a role, although larger studies are needed.

Dacryocystorhinostomy is the procedure to restore the tear pathway. The Italian Addeo Toti first introduced the external approach in 1904, which was later modified by Dupuy-Dutemps and Bourguet [11]. Internal DCR, on the other hand, pioneered by Caldwell in 1893 and improved by McDonogh and Meiring in 1989, is the preferred treatment method at the moment. The initial methodology involves the opening of the lacrimal sac on the lateral wall, anterior to the middle concha, the creation of a mucosal flap, and subsequent marsupialization of the lacrimal sac [13,14]. Endoscopic endonasal DCR has many advantages, mainly no external scar and less chance of injury to adjacent structures, such as the angular vein [9]. Endonasal endoscopic dacryocystorhinostomy is usually a successful treatment procedure with results comparable to those of the traditional external dacryocystorhinostomy [15-17]. Also, it has been described as an effective procedure in pediatric patients, although none were included in this study [18].

In this series, 88.9% of the patients were treated endoscopically due to an absence of specific indications for external DCR, given the described benefits, as well as patient preferences. Significantly, septoplasty was not necessary in any of the cases. The results show that the incidence of re-stenosis after endoscopic DCR is approximately 8,8%. Retrospectively, considering the presence of nasal septal deviation to some degree in all patients, it may be a factor hindering the wide opening of the lacrimal sac and subsequent re-stenosis. Interestingly, no subsequent re-stenosis was observed after stent placement due to re-stenosis without septoplasty. The placement of a stent is controversial in the literature, with some authors concluding that it may increase the incidence of stenosis, while others report no difference in the success rate [19-21]. The cases in which a silicone stent was placed in this series were successful, although the series is small. In regards to external DCR, no conclusion can be reached regarding the incidence of re-stenosis following this procedure. Studies with a larger number of included patients are needed to accurately determine the incidence of re-stenosis following these procedures.

Multiple factors are most likely to play a role in the incidence of DC, and a multi-disciplinary approach is key to the diagnosis and treatment, with ophthalmologists having an important role in the differential diagnosis of epiphora. This study reaffirms the successful results of endoscopic dacryocystorhinostomy in the treatment of dacryocystitis, although questions arise as to the role of superior nasal septal deviation and the importance of septoplasty in the success of DCR. The limitations of this study include its retrospective nature and single-institution experience. Multi-institutional prospective studies aimed specifically at superior nasal septal deviation in the context of dacryocystitis would significantly contribute to the literature.

## Conclusions

Dacryocystitis is a disease caused by an obstruction of the nasolacrimal duct, leading to an over-accumulation of tears in the lacrimal sac and subsequent inflammation. Although multiple factors may play a role in the disease development, the superior nasal septal deviation is an emerging factor both for the incidence of dacryocystitis and the development of re-stenosis after dacryocystorhinostomy. Further studies are needed to find the exact types of nasal septum deviations carrying the greatest risk for disease development, as well as the role of septoplasty in the result of treatment.
